# Eugenics and Preventive Medicine

**Published:** 1912-12

**Authors:** 


### Department of Eugenics.

Conducted by Dr. Malone Duggan and Dr. Theo. Y. Hull.

### THE TEXAS STATE SOCIETY OF SOCIAL HYGIENE.

Headquarters: San Antonio, Texas.

DR. MALONE DUGGAN, President, San Antonio.
DR. F. E. DANIEL, 1st Vice President, Austin.
PROF. A. CASWELL ELLIS, 2d Vice President, Austin.
MRS. ELI HERTZBERG, 3d Vice President, San Antonio.
DR. THEO. Y. HULL, Secretary, San Antonio.
MR. W. L. EVANS, Treasurer, San Antonio.

EXECUTIVE COMMITTEE.

Dr. Malone Duggan, Chm.
Dr. Theo. Y. Hull, Sec'y.
Dr. W. F. McCaleb.
Hon. C. H. Jenkins.
Rev. A. W. S. Garden.
Hon. J. C. Willacy.
Mr. W. L. Evans.
Dr. Frank D. Boyd.
Dr. J. R. Nichols.
Dr. J. M. McCutchan.
Dr. W. A. King.
Dr. J. W. Torbett.
Dr. F. C. Walsh.
Dr. Joe Reuss.
Dr. Frank Paschal.

### Eugenics and Preventive Medicine.

Modern sanitation has been the greatest single blessing to mankind. It has made possible the splendid achievements of our social progress. Without it there could not be the physical condition on which normal progress depends. If we have greater inventions, greater utilities, greater commerce, greater conveniences and greater humanitarianism than any civilization preceding us, it is due directly to modern sanitary methods. Not alone has it added fifteen years to the span of human life, and averted incalculable pain and sorrow, but it is responsible, more than any other factor, for the evolution of the moral spirit that prompts humanity to greater endeavor. But, withal, has it been an unmitigated blessing? Has it added anything to the improvement of the innate man? Has the quality of man improved in the last two thousand years? If we accept modern biological investigations, there is no evading the answer. The fact is that man not only has not improved intrinsically, but in reality is degenerating. If this be true, why? For, as Saleeby says: "There is no wealth but life, and if the inherent quality of life fails, neither battleships, nor libraries, nor symphonies, nor free trade, nor tariff reform, nor anything else, will save a nation." The reason for it is not far to find. Under bad conditions of sanitation, the death rate was high, with a correspondingly high birth rate. Under modern conditions, both the birth rate and death rate are lower, but with this difference—when natural selection prevailed the worst types perished, whereas, under man-made regulations the worst types survive, until we find the unfit increasing at a greater rate than in the normal, gifted classes. This does not mean that we would recall the old days of squalor and death. It simply emphasizes the fact that our "sense of social responsibility has become our racial responsibility." Sanitation has been but a link in the chain of evolution. We now must go a step further and deliberately control the quality of the race by permitting none but the fit to procreate. Thus the eugenist joins hands with the sanitarian for the betterment of the race, but with a more altruistic purpose, e. g., regeneration, viz., social environment.

